# The Structure of an LIM-Only Protein 4 (LMO4) and Deformed Epidermal Autoregulatory Factor-1 (DEAF1) Complex Reveals a Common Mode of Binding to LMO4

**DOI:** 10.1371/journal.pone.0109108

**Published:** 2014-10-13

**Authors:** Soumya Joseph, Ann H. Kwan, Philippa H. Stokes, Joel P. Mackay, Liza Cubeddu, Jacqueline M. Matthews

**Affiliations:** 1 School of Molecular Bioscience, University of Sydney, Sydney, NSW, Australia; 2 School of Science and Health, University of Western Sydney, Campbelltown, NSW Australia; The National Institute of Diabetes and Digestive and Kidney Diseases, United States of America

## Abstract

LIM-domain only protein 4 (LMO4) is a widely expressed protein with important roles in embryonic development and breast cancer. It has been reported to bind many partners, including the transcription factor Deformed epidermal autoregulatory factor-1 (DEAF1), with which LMO4 shares many biological parallels. We used yeast two-hybrid assays to show that DEAF1 binds both LIM domains of LMO4 and that DEAF1 binds the same face on LMO4 as two other LMO4-binding partners, namely LIM domain binding protein 1 (LDB1) and C-terminal binding protein interacting protein (CtIP/RBBP8). Mutagenic screening analysed by the same method, indicates that the key residues in the interaction lie in LMO4_LIM2_ and the N-terminal half of the LMO4-binding domain in DEAF1. We generated a stable LMO4_LIM2_-DEAF1 complex and determined the solution structure of that complex. Although the LMO4-binding domain from DEAF1 is intrinsically disordered, it becomes structured on binding. The structure confirms that LDB1, CtIP and DEAF1 all bind to the same face on LMO4. LMO4 appears to form a hub in protein-protein interaction networks, linking numerous pathways within cells. Competitive binding for LMO4 therefore most likely provides a level of regulation between those different pathways.

## Introduction

LMO4 is a member of the LIM-only protein family (LMO1–4) of metazoan transcriptional co-regulators, and has important roles in neural and skeletal development [1], [2], [3]. It is overexpressed in greater than 50% of sporadic breast cancers and overexpression is correlated with a poor outcome [4], [5]. Although LMO proteins are known to regulate gene transcription, they do not bind DNA directly. Rather, they use their protein-interacting LIM (Lin-11/Isl1/Mec-3) domains to make simultaneous contacts with two or more other proteins that do bind to DNA, such as GATA1 and basic helix-loop-helix (bHLH) proteins [6], [7]. Interaction of these LIM domains with the adaptor protein LIM domain binding protein 1 (LDB1/CLIM2/NLI) facilitates long range chromatin interactions (e.g., [8], [9], [10], [11]) via the self association of LDB1 [12], [13], [14].

Whereas LMO1–3 have relatively restricted expression patterns and can sometimes substitute for each other [15], LMO4, the most divergent member of the LMO family, is expressed much more broadly and appears to have a correspondingly broader range of interaction partners. Reported binding partners include LDB1 [16], [17], GATA6 [18], the tumour suppressor CtIP/RBBP8 [19], [20], [21], the bHLH proteins HEN1 [22] and neurogenin-2 [23], estrogen receptor-α [24], the membrane receptor protein neogenin [25], protein tyrosine phosphatase 1B (PTP1B) [26], the transforming growth factor β family member BMP7 [27], the transcription factor DEAF1 [28], and components of a nucleosome-remodelling complex (HDAC1, HDAC2 and MTA1) [24]. In line with the wide range of reported partners, LMO4 has diverse functions in normal and diseased states. During mouse embryogenesis it is involved in closing the neural tube during gastrulation [1], [3], anterior-posterior patterning [1], development of the inner ear [13], neural development [29] and sex determination [30]. In adult mice it is implicated in memory and learning [31], insulin secretion and sensitivity [26], [32], adipogenesis [33] and the development of mammary glands during pregnancy [4]. LMO4 also appears to regulate the cell cycle and can localise to centrosomes [34]. Aside from breast cancer, LMO4 is overexpressed in non-small-cell lung cancer [35]. Overexpression of LMO4 is associated with good prognosis in pancreatic cancer [36], whereas decreases in expression have been correlated with aggressive meningioma [37], hormone-refractory recurrent prostate cancer [38] and Alzheimer's disease [39], [40]. Establishing the mechanisms by which LMO4 binds its partner proteins will help us understand how LMO4 contributes to these activities, and how these activities may be connected. However, of the many known protein partners of LMO4, to date only interactions with LDB1 and CtIP have been physically characterised.

The LIM-interacting domain (residues 300–330) of LDB1 (LDB1_LID_), which is intrinsically disordered in isolation, forms β-zippers that augment the β-hairpins in each LIM domain [41] of the tandem LIM domains of LMO4 (LMO4_LIM1+2_). The proteins bind each other in a head-to-tail manner, that is, with the C-terminus of one protein proximal to the N-terminus of the other [42], [43], [44]. An analogous complex is formed between the first LIM domain from LMO4 (LMO4_LIM1_) and residues 664–674 of CtIP_641–685_, which also appears to be intrinsically disordered in isolation [19]. LMO4•LDB1, LMO4•DEAF1 and etc. are used herein to designate engineered tethered complexes in which “•” represents a Gly/Ser linker. The name order reflects the order of the domains in the construct. The structures of LMO4_LIM1_•LDB1 and CtIP_664–674_•LMO4_LIM1_ overlay well; it is clear that LDB1_LID_ and CtIP_664–674_ bind LMO4_LIM1_ in an identical manner, despite low sequence identity. These overlapping binding sites and other experimental data indicate that the binding of CtIP or LDB1 to LMO4 is mutually exclusive and, when all are present in the same location, CtIP and LDB1 must compete for binding to LMO4 [19].

DEAF1 exhibits many biological parallels with LMO4, suggesting that the reported interaction has important biological roles. Both proteins contribute to anterior-posterior patterning and neural tube closure in the developing mouse [1]. Like LMO4, DEAF1 is involved in insulin signalling [45], onset of Type 1 diabetes [46], cognitive [47], [48] and mood disorders [49]. They are co-expressed in multiple cell types [17], [50] and have similar knock-in or knock-out phenotypes. Further, overexpression of either protein enhances the proliferation of breast epithelial cells [4], [51].

We previously reported that LMO4 binds a putatively disordered region in DEAF1 (DEAF1_404–438_). This region of DEAF1 lies close to a coiled-coil domain that forms tetramers in vitro [52], and encompasses the nuclear export signal (NES) [53]. In addition, we conducted cell-based localisation studies using constructs spanning the LMO4-interacting domain, NES and coiled-coil domains of DEAF1 to show that LMO4 likely modulates the sub-cellular localisation of DEAF1. Here we address the molecular basis for complex formation by LMO4 and DEAF1. We show using the yeast two-hybrid assay that both LIM domains of LMO4 are required for the interaction, and that the LIM2 domain of LMO4 (LMO4_LIM2_) and the N-terminal region from DEAF1_404–438_ are the major determinants of binding. We generated a stable complex comprising these domains, and determined its solution structure. The structure reveals that DEAF1 binds LMO4_LIM2_ in an extended head-to-tail conformation, contacting the same face on LMO4 as LDB1, and demonstrates that a common mode of binding to LMO4 exists for DEAF1, CtIP and LDB1. Our data suggest that competition for binding to LMO4 is a common feature of LMO4-binding partners, and over- or under-expression of LMO4 can disrupt multiple networks of interactions within cells to promote disease.

## Materials and Methods

### Cloning

Residue numbering refers to mouse LMO4 and DEAF1 (NCBI accession numbers: **NP_001155241** and **NM_016874**, respectively). pGBT9 yeast two-hybrid plasmids encoding LMO4, LMO4_LIM1_ and LMO4_LIM2_, and pGBT9-LMO4•LDB1 were described previously [19], [43]. A yeast two-hybrid plasmid encoding DEAF1_45–566_ was a gift from Jane Visvader. Vectors containing inserts encoding surface mutants of LMO4 [19] were sub-cloned into pGAD10. DEAF1 mutants were generated using overlap extension PCR on the background template DEAF1_404–438_457–479_ [52]. L4-DEAF1 (DEAF1_404–438_ containing a T435D mutation and a polyproline C-terminal tail) was cloned into the plasmid pRSET-A.

### Yeast two-hybrid assays

Yeast two-hybrid assays were conducted as described previously [43]. *Saccharomyces cerevisiae* strain AH109 (Clontech) were co-transformed and plated on solid media lacking leucine and tryptophan (–L/–W; growth). Liquid cultures of co-transformed yeast were serially diluted (A_600 nm_ = 0.2, 0.02 and 0.002) and spotted (2 µL) on plates that also lacked histidine (−L/−W/−H; low stringency), and included 0.5 mM 3-amino-1,2,4,-triazol (−L/−W/−H + 3-AT; medium stringency), or excluded adenine (−L/−W/−H/–A; high stringency), as well as growth control plates.

### Production of recombinant protein

The tethered complexes LMO4_LIM2_•DEAF1_404–418_, and DEAF1_404–418_•LMO4_LIM2_ were produced as described previously for LMO4_LIM2_•DEAF1_404–418_ [54]. In these two constructs LIM2 corresponds to LMO4_77–147_ and LMO4_83–147_, respectively. L4-DEAF1 was produced as a hexahistidine-tagged construct in *Escherichia coli* expression strain Rosetta 2 (Novagen). Cells were cultured in rich media or, for isotopic labelling, in minimal media containing ^15^NH_4_Cl as the sole nitrogen source. Expression was induced with 1 mM isopropyl-β-D-thiogalacto-pyranoside (IPTG) at 37 °C for 4 h. The cells were lysed under denaturing conditions in buffer A (20 mM Tris-base at pH 8.0, 150 mM NaCl and 20 mM imidazole) containing 8 M guanidine-HCl. Cleared lysate was incubated with Ni-NTA resin for 1 h at 4 °C. The resin was washed with buffer A containing 6 M urea and then with buffer A containing 2 mM CaCl_2_. The peptide was treated with thrombin on resin overnight at room temperature and eluted with buffer A. The acidified eluate was applied to a preparative C18 reversed-phase HPLC column. A gradient of acetonitrile was applied over a background of 0.1% TFA in MilliQ-water. The protein was lyophilised, and redissolved in buffers as required.

### NMR experiments and structure determination

Chemical shifts for LMO4_LIM2_•DEAF1_404–418_, in 20 mM sodium acetate at pH 5.0, 35 mM NaCl, 0.5 mM TCEP-HCl, 34 µg mL^–1^ chloramphenicol and Complete EDTA-free protease inhibitor (one tablet per 50 mL), were assigned using standard triple resonance NMR experiments as described in [54]. Distance restraints were obtained from ^1^H-^1^H 2D NOESY, ^15^N-edited NOESY and ^13^C-edited NOESY spectra, all with mixing times of 150 ms. NOE peaks were manually picked, checked and corrected where necessary. The ensemble of structures for LMO4_LIM2_•DEAF1_404–418_ was calculated using ARIA 1.2 implemented in CNS 1.21 [55]. Default parameters were used except where stated. The number of molecular dynamics steps was: initial stage, 40000; refinement stage, 8000; first cooling step, 40000; and second cooling step, 8000. The upper limit for NOE distance estimates was increased by 0.15 Å from the default value. A mixing time of 150 ms and a rotational correlation time of 5.16 ns were used to set relaxation matrix parameters. A zinc patch was included to define zinc co-ordination geometry (Table 1) based on the coordinates of the LIM2 domains from LMO4 and LMO2 in the LMO_LIM1+2_•LDB1_LID_ (PDB IDs: **1RUT** and **2XJY**, respectively. **File S1**). In the first iteration 200 structures were calculated, with 20 structures calculated for each of the 7 intermediate iterations, and 600 structures in the final iteration. The 50 lowest energy structures from the final iteration were further refined in a shell of water using the standard ARIA protocol. Longitudinal (*T*_1_), transverse (*T*_2_) and heteronuclear NOE relaxation experiments were performed on 600 µM ^15^N-labelled LMO4_LIM2_•DEAF1_404–418_ using the Bruker pulse programs hsqct1etf3gpsi3d, hsqct2etf3gpsi3d and hsqcnoef3gpsi3d, respectively. The relaxation delays used for measuring ^15^N-*T*_1_ time constants were 0.1, 0.15, 0.2, 0.3, 1, 1.4, 1.5 and 2.2 s and those for measuring *T*_2_ time constants were 17, 34, 51, 68, 85, 102, 136, 153, 170, 221 and 255 ms. The same relaxation experiments were recorded at 800 MHz except that the *T*_2_ relaxation delays at 800 MHz were 16, 32, 48, 64, 80, 96, 128, 144, 160, 208 and 240 ms. Lipari-Szabo ordered parameters (S^2^) were calculated using the model-free module (fully automated mode) in relax [56], [57].

**Table 1:** NMR restraints and refinement statistics for LMO4_LIM2_DEAF1_404–418_.

**Distance restraints**		
Total NOE	894	
Ambiguous	92	
Intra-residue	450	
Sequential (|i−j| = 1)	172	
Medium-range (|i−j|<5)	45	
Long-range (|i−j|≥5)	227	
S-Zn (2.3 Å)^a^	6	
N-Zn (2.0 Å)^a^	1	
O-Zn (2.03 Å)^a^	1	
*Total dihedral angle restraints (TALOS)* a		
φ	43	
ψ	43	
*Zinc(II) angle restraints* b		
S-Zn-S (112°)	6	
C-S-Zn (107°)	6	
S-Zn-N (107°)	3	
C-N-Zn (125°)	1	
S-Zn-O (102°)	3	
C-O-Zn (125°)	1	
*Atomic RMSD (Å)*	*Backbone*	*Heavy*
LMO4_LIM2_•DEAF1_404–418_ c	0.691	1.102
LMO4^c^	0.666	1.102
DEAF1^c^	0.506	0.891
*PROCHECK–Ramachandran Statistics (all)*		
Most Favoured (%)	73.3	
Additionally allowed (%)	23.1	
Generously allowed (%)	2.2	
Disallowed (%)	1.4	
*Mean deviations from the ideal geometry*		
Bond Lengths (Å)	0.00390±0.00023	
Bond Angles (°)	0.493±0.018	
Impropers (°)	1.35±0.11	
dDistance violations >0.5 Å	2	

a There were no dihedral angle violations >5°.

b Full parameter and topology files are included in File S1.

c Regions of LMO4 between residues 86–139 and of DEAF1 between residues 404–414 including S208 of the glycine-serine linker were considered to be structured because the residues contained within had sum of angle order parameters (φ + ψ)>1.8 except for residues 103–105 of LMO4 and residues 404, 406 and 407 of DEAF1.

d Distance violations were restricted to disordered regions of the protein.

Recycle delays of 4 s were used in these experiments. Integrated peaks were fitted to two-parameter exponentials using the relaxation analysis module in SPARKY [58]. ^1^H-^15^N heteronuclear NOEs were calculated by taking the ratio of cross-peak intensities with and without proton saturation during relaxation delays. One-dimensional ^1^H and two-dimensional ^15^N-HSQC spectra of L4-DEAF1 were performed in 20 mM sodium acetate at pH 5.0 and 35 mM NaCl. Images of structures were generated using PyMol, simple homology models were generated using SWISS-MODEL [59] or mutation of residues in PyMol, and the surface area of the LMO4-DEAF1 interface was calculated using PISA [60].

### Multi-angle laser-light scattering (MALLS)

Purified proteins were subjected to size-exclusion chromatography using a Superose-12 10/30 size-exclusion column (GE Healthcare) with an in-line MiniDawn MALLS detector (Wyatt Technology) and Wyatt Refractometer. Proteins were eluted in 20 mM Tris-acetate at pH 8.0, 50 mM NaF and 0.5 mM TCEP-HCl using a flow rate of 0.5 mL min^−1^. The weight-average molecular weight was calculated using the intensity of scattered light in combination with the change in refractive index. Protein concentration at the detector was determined by the change in refractive index.

### Far UV-circular dichroism (CD) spectropolarimetry

The far-UV CD spectrum of L4-DEAF1 (40 µM) buffered in 20 mM Tris-acetate and 50 mM NaF was recorded on a Jasco J-720 spectropolarimeter at 20°C in a 1-mm quartz cuvette. The spectrum represents the average of three accumulations collected at a rate of 20 nm min^−1^. Data were collected at a resolution of 0.5 nm and smoothed over a moving average of five consecutive data points. Curves were buffer-baseline corrected.

### Accession numbers

The coordinates of the 20 lowest energy water-refined structures of LMO4_LIM2_•DEAF1_404–418_ have been deposited in the PDB with PDB ID: **2MBV**. The NMR assignments were previously deposited in the BMRB (deposition number **18898**) [54].

## Results

### Identification of domains and residues on LMO4 important for binding to DEAF1

**Figure 1:**
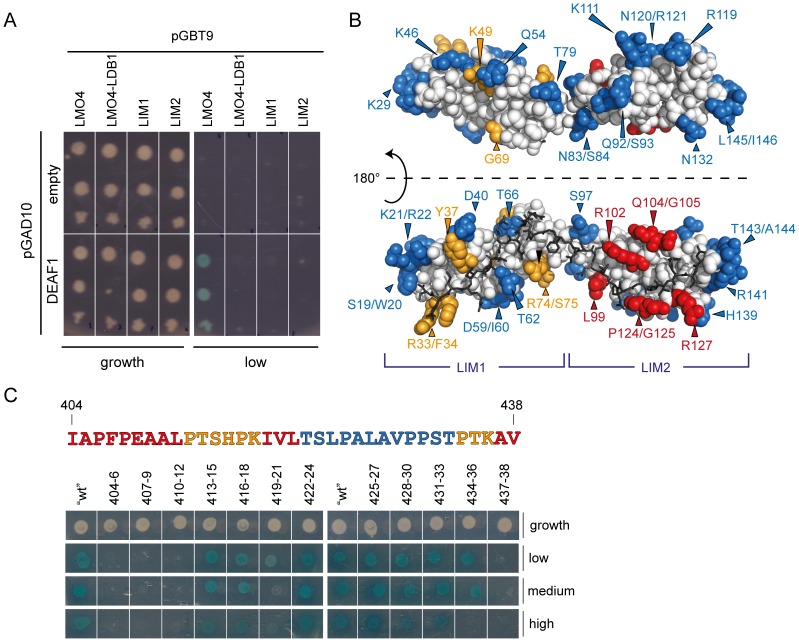
Yeast two-hybrid and mutagenic analysis of LMO4-DEAF1 binding. (A) Data showing the interaction of DEAF1_45–566_ (DEAF1) or control (empty) with the tandem LIM domains of LMO4 (LMO4), the isolated LIM domains of LMO4 (LIM1 and LIM2) or a pre-formed LMO4•LDB1_LID_ complex. These were spotted onto low-stringency interaction plates or growth control plates. “Empty” refers to pGAD10 vector lacking an insert. (B) Summary of yeast two-hybrid work. Surface residues of LMO4 that when mutated strongly affected (red), attenuated (orange) or had no effect (blue) on interaction with DEAF1 are mapped onto the structure of LMO4•LDB1_LID_ (1RUT). Non-mutated residues are in white, and LDB1_LID_ is shown as dark sticks. (C) Mutagenic scanning of the minimal LMO4-binding domain of DEAF1 (in the DEAF1_404–438_457–479_ construct). Residues in DEAF1_404–438_ were systematically mutated to alanine or glycine in sets of three (or two) as indicated and analysed for binding to LMO4 using yeast two-hybrid assays. Co-transformants were spotted onto selective media (low, medium and high stringency plates) as well as growth control plates. The sequence of DEAF1 is coloured according to whether the mutation strongly affected binding (red), attenuated binding (orange) or had no effect (blue) compared to wild-type positive control on each plate (“wt”). Thick white lines indicate separate plates.

Yeast two-hybrid assays were used to test the interaction of DEAF1_45–566_ with isolated LIM domains of LMO4 (LIM1 and LIM2). LMO4 was expressed either as a construct containing one or both LIM domains (LMO4) or as a fusion protein with LDB1_LID_ which makes an ‘intramolecular complex’ (LMO4•LDB1_LID_). An interaction between LMO4 and DEAF1 was only observed in the presence of both LIM domains and in the absence of LDB1_LID_ (**Fig. 1a**). These data suggest that both LIM domains are involved in binding DEAF1 and that the presence of LDB1_LID_ prevents DEAF1 from binding to LMO4. Thus, DEAF1 and LDB1 share similar binding faces on LMO4, or the presence of LDB1 induces a conformational change in LMO4 that prevents DEAF1 binding.

To identify the DEAF1 interaction surface on LMO4, a library of LMO4 surface mutants was screened for an interaction against DEAF1_45–566_ using yeast two-hybrid assays (**Fig. 1b**). The interaction was considered to be strongly affected if no growth was observed on medium stringency selection plates (red residues), and attenuated if growth on these plates was less than that observed for wild-type LMO4 (orange residues). In general, the residues for which the interaction was disrupted lie predominantly on the LDB1-binding face of LMO4 (**Fig. 1b**), supporting the idea that a common binding face on LMO4 exists for DEAF1 and LDB1. Mutated residues for which the interaction was most strongly affected (i.e., L99, R102, Q104/G105, P124/G125 and R127) are located on LMO4_LIM2_, whereas those for which the interaction was attenuated (R33/F34, Y37, K49, G69 and R74/S75) are part of LMO4_LIM1_ (**Fig. 1b**). Thus, although both LIM domains of LMO4 are required for the interaction with DEAF1 to be detected in yeast, the second LIM domain (LIM2) appears to more important for binding.

### Characterisation of the LMO4-interaction domain in DEAF1

We previously defined the LMO4-interaction domain in DEAF1 as DEAF1_404–438_ [52], which is predicted to be unstructured in the context of the full-length protein [52], [53] by sequence analysis programs that predict order/disorder, PONDR [61] and IUPred [62], and lacks substantial levels of secondary structure by JPred3 (which predicts secondary structure based on sequence) [63]. To identify residues of DEAF1 that are important for binding LMO4, sequential sets of three residues in DEAF1_404–438_ were mutated to alanine (or glycine if the original residue was alanine) and screened for an interaction with LMO4 by yeast two-hybrid analysis (**Fig. 1c**). In general, mutations to the N-terminal half (404–421) of DEAF1_404–438_ strongly affected the interaction, whereas mutations to the C-terminal half had little effect.

**Figure 2:**
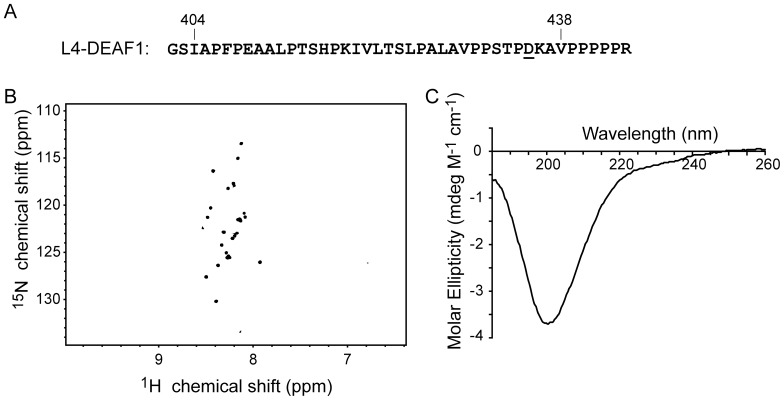
The LMO4-binding domain from DEAF1 is disordered in solution. (A) The sequence of L4-DEAF1 includes residues 404–438 of DEAF1 (bold), a T435D point mutation (underlined) and a polyproline C-terminal tail (PPPPPR). The two N-terminal residues (GS) are an artefact of the plasmid and remain after treatment with thrombin. (B) ^15^N-HSQC spectrum of L4-DEAF1 (160 µM) was recorded in 20 mM sodium acetate at pH 5.0 and 35 mM NaCl at 298 K on a 600 MHz spectrometer equipped with a TCI-cryogenic probehead. (C) The far-UV CD spectrum of L4-DEAF1 (40 µM) dissolved in 20 mM Tris-acetate at pH 8.0 and 50 mM NaF.

We could not produce a recombinant peptide corresponding to DEAF1_404–438_ in *E. coli*. The peptide was either poorly expressed or was rapidly degraded. Instead, we expressed and purified L4-DEAF1, a construct of DEAF1_404–438_ that contains a point mutation, T435D (originally generated as a pseudo-phosphorylation mutant for a separate study), and a polyproline tail that was added to the C-terminus to enhance proteolytic stability [64], [65] (**Fig. 2a**). NMR experiments and far-UV CD spectropolarimetry were used to assess the fold of L4-DEAF1. The ^15^N-HSQC spectrum shows sharp peaks that cluster between 8–8.5 ppm in the ^1^H dimension (**Fig. 2b**). This type of poor dispersion of proton resonances is a hallmark of intrinsically disordered proteins [66]. The far-UV CD spectrum is also characteristic of a largely disordered peptide, with a minimum at ∼200 nm and negative signal at 195 nm (**Fig. 2c**). Thus, DEAF1_404–438_ is intrinsically disordered in isolation.

### Designing an LMO4-DEAF1 complex

**Figure 3:**
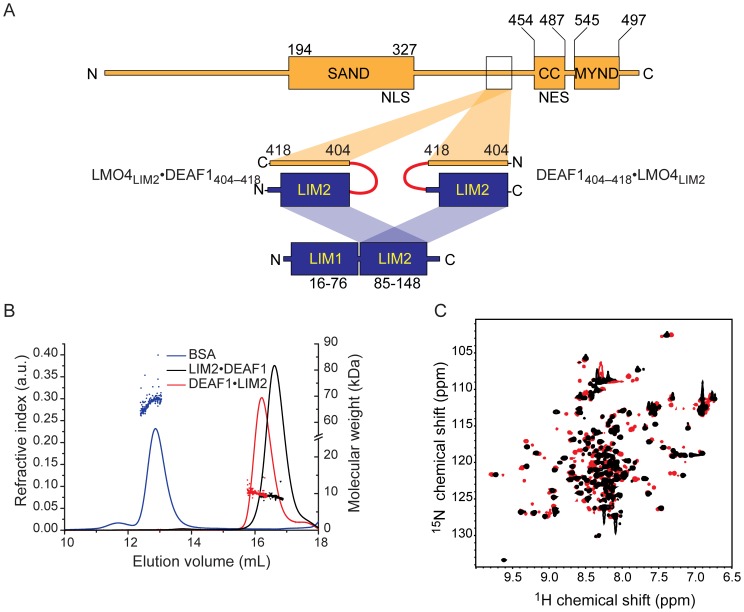
Engineering tethered LMO4_LIM2_•DEAF1_404–418_ and DEAF1_404–418_•LMO4_LIM2_ complexes. (A) Schematics of full-length LMO4 (blue) and DEAF1 (orange) and engineered ‘intramolecular complexes’ of LMO4_LIM2_ and DEAF1_404–418_. The complexes are tethered via a glycine-serine linker (red) either from the C-terminus of LMO4 to the N-terminus of DEAF1 or vice versa. SAND, coiled-coil (CC) and MYND domains, and nuclear localisation (NLS) and nuclear export (NES) signals in DEAF1 and the LIM1 and LIM2 domains in LMO4 are indicated. (B) MALLS analysis of tethered constructs as indicated; protein concentrations at the detectors are 30 µM. Lines represent the refractive index and calculated molecular weights are shown as symbols. Monomeric BSA (blue) was used as a standard. (C) ^15^N-HSQC spectra of LMO4_LIM2_•DEAF1_404–418_ (black) and DEAF1_404–418_•LMO4_LIM2_ (red) were recorded in 20 mM sodium acetate at pH 5.0, 35 mM NaCl and 0.5 mM TCEP-HCl at 298 K on a 600 MHz spectrometer.

Recombinant forms of LMO4 including either or both LIM domains tend to be poorly soluble and/or are aggregation prone unless expressed as a tethered complex in which an interacting peptide domain from LDB1 or CtIP is tethered to LMO4 via a flexible linker [19], [43]. We used the same strategy to engineer LMO4•DEAF1 complexes. A range of different LMO4•DEAF1 complexes were generated that contained both LIM domains of LMO4 and DEAF1_404–438_. Although some of these complexes showed promise in terms of soluble expression and preliminary structural characterisation (**Fig. S1** in **File S2**), they were not sufficiently stable for detailed structural characterisation. In particular, these proteins were prone to proteolytic cleavage at K418 of DEAF1, as determined by mass spectrometry (Sydney University Proteomics Research Unit; SUPRU). A K418Q mutant of LMO4•DEAF1_404–438_ was generated in an effort to prevent proteolytic degradation, but this protein was insoluble. Given that our yeast two-hybrid data indicate that binding is mainly mediated by LMO4_LIM2_ and the N-terminal half of DEAF1_404–438_, we generated protein constructs that included only these domains. We engineered these complexes in both orientations (LMO4_LIM2_•DEAF1_404–418_ and DEAF1_404–418_•LMO4_LIM2_, where the order of the domains in the name corresponds to the order in the construct; **Fig. 3a**). Both tethered complexes were subjected to size-exclusion chromatography in combination with multi-angle laser-light scattering (SEC-MALLS). For both constructs the theoretical molecular weights (LMO4_LIM2_•DEAF1_404–418_ = 10.1 kDa and DEAF1_404–418_•LMO4_LIM2_ = 9.3 kDa) and observed experimental molecular weights (LMO4_LIM2_•DEAF1_404–418_ = 10.7±0.8 kDa and DEAF1_404–418_•LMO4_LIM2_ = 10.1±0.6 kDa) were in excellent agreement, indicating that the proteins are predominantly monomeric (**Fig. 3c**). The ^15^N-HSQC spectrum of LMO4_LIM2_•DEAF1_404–418_ was of high quality, with most peaks exhibiting similar intensities, in contrast to the broad range of signal intensities observed in the ^15^N-HSQC of DEAF1_404–418_•LMO4_LIM2_ (**Fig. 3b**).

### Determining the structure of the second LIM domain of LMO4 in complex with DEAF1

**Figure 4:**
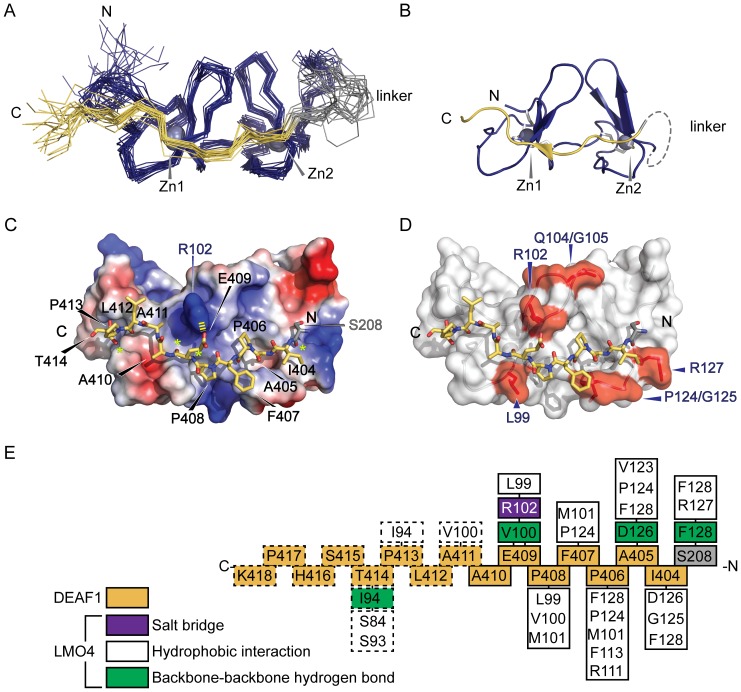
The structure of LMO4_LIM2_•DEAF1_404–418_. (A) Overlay of the 20 lowest energy structures. Backbone regions from LMO4_LIM2_ (blue), linker (grey) and DEAF1 (yellow) are shown as lines with only Cα atoms shown. Zinc ions (Zn1 and Zn2) appear as grey spheres. (B) Ordered regions of the lowest energy structure are shown as ribbons, coloured as in panel (A). Side-chains of zinc-coordinating residues are shown as sticks. The linker is represented as a dashed grey line. (C) LMO4_LIM2_ is represented as an electrostatic surface. Positively and negatively charged surfaces are coloured blue and red, respectively. DEAF1 is shown as orange sticks except for nitrogen (blue) and oxygen (red) atoms. Residues of DEAF1 are labelled. The N- and C-termini of DEAF1 are marked. The salt bridge formed between R102 of LMO4 and E409 of DEAF1 is denoted by a dashed yellow line. (D) Surface mutations of LMO4 that abrogated (red) its interaction with DEAF1 (yellow sticks) in yeast two-hybrid assays are mapped onto the lowest energy solution structure of LMO4_LIM2_•DEAF1_404–418_. (E) Summary of interactions at the LMO4-DEAF1 interface. Residues from LMO4_LIM2_ are positioned directly above or below their interaction partners from DEAF1_404–418_ (orange) and coloured according to the type of interaction. S208 (grey) of the linker makes contacts in more than half the structures in the final ensemble. Residues S208 from the linker and I404–A410 from DEAF1 and their interactions are indicated with solid lines. Remaining DEAF1 residues and interactions are indicated with dashed lines.

The NMR spectra of LMO4_LIM2_•DEAF1_404–418_ were assigned as described previously [54] and the structure was determined using standard solution NMR methods. The structured regions of the complex are LMO4_86–139_ and DEAF1_404–414_ (**Table 1** and **Fig. 4**). The r.m.s.d. of these regions in the ensemble is 0.7 Å for backbone atoms and 1.1 Å for all heavy atoms. The structure of LMO4_LIM2_ is typical of LIM domains [67], which contain two zinc-binding modules, each of which comprises two orthogonally arrayed β-hairpins followed by a short helical region of variable length. In this case the α-helices are short and poorly defined. The first β-hairpin and helix in each zinc-binding module coordinate one zinc ion. The first zinc-ion is coordinated by C87, C90, H109 and C112, and the second by C115, C118, C137 and D140 (**Fig. 4b**). A hydrophobic core is formed by residues from the first (M101, A103, Q104, Y108 and F113) and second (L122, F54 and Y56A) zinc-binding modules packing against each other. Apart from a single residue preceding the N-terminus of DEAF1 (S208), the glycine-serine linker between LMO4_LIM2_ and DEAF1_404–418_, appears to be disordered (**Table 1**).

The DEAF1 peptide binds in an extended, head-to-tail conformation across the length of LMO4_LIM2_ (**Fig. 4a–d**), indicating that this motif, like other LIM-interacting domains, becomes structured upon binding. The interface between LMO4_LIM2_ and DEAF1_404–418_ buries a surface area of ∼1700 Å^2^ and complex formation appears to be mediated by hydrophobic interactions between side-chains (**Fig. 4**). The side-chain of residue DEAF1_A405_ is buried in hydrophobic core of LMO4_LIM2_ between the two zinc-binding modules, and a number of other residues, particularly in the stretch I404–E409, make surface hydrophobic contacts. In at least half the conformers in the ensemble, four backbone-backbone hydrogen bonds are formed between LMO4_LIM2_ and DEAF1 (**Fig. 4e**), creating short segments of β-strand that augment β-hairpins in LMO4. A single salt bridge formed between DEAF1_E406_ and LMO4_R102_ (**Fig. 4c and 4e**) is seen in more than half the structures in the ensemble and may help define the binding register.

### The effect of the tether on the structure of LMO4_LIM2_•DEAF1_404–418_

**Figure 5:**
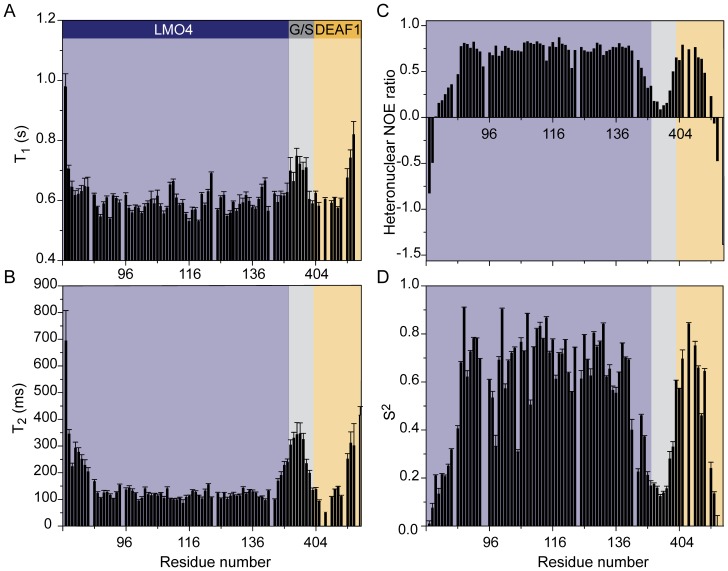
Relaxation analysis of LMO4_LIM2_•DEAF1_404–418_. (A) Longitudinal (*T*_1_), (B) transverse (*T*_2_) relaxation time constants, (C) heteronuclear NOEs, calculated as the ratio of peak intensities with and without proton saturation, all at 600 MHz. (D) Lipari-Szabo (S^2^) parameters for each assigned backbone amide group in LMO4_LIM2_•DEAF1_404–418_ calculated from data recorded at 600 MHz and 800 MHz, using the program relax. Error bars represent one standard deviation from the curve fit for each residue. Background colours indicate regions belonging to LMO4 (blue), DEAF1 (yellow) or the glycine-serine linker (G/S; grey).

The use of a tether can place steric restraints on complex formation. We previously showed that chemical shift data is consistent with disorder in the linker [54]. Here we used ^15^N-NMR relaxation data to assess whether the tether might introduce strain into the complex, and also to examine the overall dynamics of the complex. The data indicate that the N- and C-termini and the glycine-serine linker between LMO4_LIM2_ and DEAF1_404–418_, with the exception of S208, undergo substantial motion, implying that the linker is flexible and is unlikely to be inducing non-native interactions (**Fig. 5**). The relaxation data show localised excursions within the structured region of LMO4 (A86–H139), which generally correspond to loop regions, such as N120–V123. In addition, the T_1_ data for two regions that form short α-helices in the crystal structure of LMO4•LDB1 (H109–K111 and E138–D140) indicates relatively dynamic structure, suggesting that these short helices are transient in solution. Low values of the order parameter S^2^ are also observed for several residues within the LIM domain. These values likely reflect local dynamics; for example, E98 and G105, which lie in a loop and a β-turn, respectively, exhibit low S^2^ values. Collectively these data may report intrinsic flexibility within LIM domains (e.g., [68], [69], [70]).

## Discussion

### The LMO4_LIM2_-DEAF1 interaction

Although both LIM domains of LMO4 were required to detect an interaction with DEAF1 in yeast two-hybrid experiments, it was only possible to determine the structure of a smaller LMO4_LIM2_•DEAF1_404–418_ tethered complex. However, comparison of ^15^N-HSQC spectra from an LMO4_LIM1+2_•DEAF1 complex and LMO4_LIM2_•DEAF1_404–418_ recorded under identical conditions shows high levels of overlap, consistent with conservation of structure for LMO4_LIM2_ and DEAF1 in both constructs (**Fig. S2** in **File S2**). The LMO4_LIM2_ in this structure is almost identical to that from the crystal structure of LMO4_LIM1+2_•LDB1_LID_ (LMO4_86–139_ from the single molecule in PDB: **1RUT** and the mean structure from PDB: **2MBV** give rise to a backbone r.m.s.d of 1.3 Å). The tether should stabilise the complex both by reducing the loss of entropy associated with binding, and by increasing the effective concentrations of interacting domains. Several pieces of data suggest that the tether allows a native-like complex between LMO4 and DEAF1. First, the data from our mutagenic interaction screens (**Table 1** and **Fig. 1**) are consistent with the solution structure of the complex. That is, the key residues from the N-terminal half of DEAF1_404–418_ and LMO4_LIM2_ are found at the LMO4_LIM2_-DEAF1 interface in the structure (**Fig. 4**). LMO4_Q104/G105_ is a minor exception; these residues form part of a β-turn that packs against LMO4_R102_, which in turn forms a salt bridge with DEAF1_E406_. Second, we have previously shown that increasing the length of the synthetic linker from eight to eleven residues, or extending the DEAF1 peptide by five residues in the N-terminal direction does not change the structure of the complex according to ^15^N-HSQC spectra [54]. In contrast, we recently showed, in a closely related system involving the LIM protein ISL1, that randomising the sequence of a LIM-binding peptide induced non-specific binding, as evidenced by significant line broadening in TROSY-^15^N-HSQC spectra [71] of the randomised peptide complex, compared to the wild-type peptide complex.

Although the LMO4_LIM2_•DEAF1_404–418_ complex appears overall to be native-like, the last residue of the synthetic linker, S208, does mediate contacts with LMO4_R127_ and LMO4_F128_ in more than half of the members of the structural ensemble (**Fig. 4e**). Similar contacts could also be mediated by DEAF1_Q403_, the residue that would replace S208 in the full sequence of DEAF1. Thus, DEAF1_Q403_, and possibly one or two additional residues from DEAF1, are also likely to be involved in the native protein interaction interface. Given the NMR data described above, these residues would be expected to slightly extend rather than significantly change the nature of the binding interface of the two proteins.

**Figure 6:**
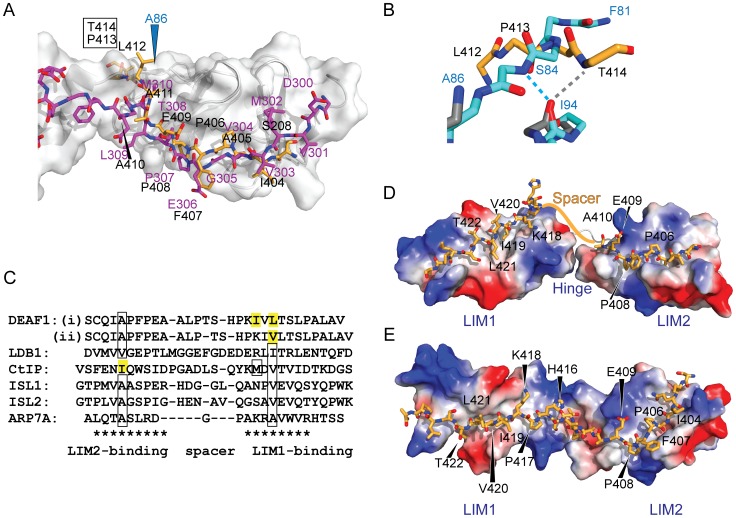
Tandem LIM LMO4-DEAF1 interactions. (A) Comparison of the lowest energy member of the LMO4-DEAF1 complex ensemble (LMO4 in grey ribbon with blue labels and DEAF1 as orange sticks with black labels) and the LMO4-LDB1 complex (PDB accession code 1RUT, LMO4 with white surface and ribbon and LDB1 in magenta). Labels for residues in DEAF1 that clash with LMO4 in the LMO4-LDB1 structure are boxed. (B) Close up of the clashing region from the previous panel, using the same colouring, but with backbone residues from LMO4-DEAF1 (grey) and LMO4-LDB1 (cyan) shown as sticks and backbone-backbone hydrogen bonds with LMO4_I94_ shown in the same colours. Only the affected residues are shown for clarity. (C) Structure-based sequence alignment of characterised LIM-peptide complexes. Residues in bold appear to be important for binding based on mutational studies, boxed residues have been shown to be buried in the hydrophobic core between the two zinc-binding modules in the relevant LIM domain, and residues highlighted in yellow are predicted to be buried based on the alignment. LIM-binding motifs are indicated with asterisks. Residues in the spacer regions are generally not conserved but are shown for completeness. Two binding registers are proposed for the LIM1-binding residues in DEAF1. (D) Simple model for binding register (i). Structures for LMO4_LIM1_-CtIP and LMO4_LIM2_-DEAF1_404–410_ were aligned over the backbone atoms of the respective LIM domains in the LMO4-LDB1 structure (1RUT), and the residues in CtIP were altered to the correspond residues in DEAF1 using the mutagenesis module in PyMol. The linker between LIM1 and LIM2 from the LMO4-LDB1 structure is shown as a white cartoon. The approximate position of DEAF1_411–415_ is indicated with an orange line. (E) Homology model for binding mode (ii) using the structure of Lhx3–Isl1 as a template. In all cases where molecules are shown as sticks, nitrogen and oxygen atoms are shown in blue and red, respectively.

Whereas the structure of the complex around the tether appears to be native-like, features near the N-terminus of LMO4_LIM2_ and C-terminus of DEAF1_404–414_ suggest that eliminating the LMO4_LIM1_ domain has had a minor effect on structure. For both this LMO4_LIM2_•DEAF1 structure and the related LMO2_LIM2_•LDB1_LID_ complex [72], the first β-hairpins in the LIM2 domains are poorly defined compared with similar complexes that also contain a LIM1 domain [6], [43], [44], [73]. This suggests that contacts between the LIM1 and LIM2 domains stabilise the structure at the N-terminus of the LIM2 domain. A comparison of the LMO4_LIM2_•DEAF1 structure with LMO4-LDB1 structures (**Fig. 6a and b**) shows that the C-terminus of the DEAF1 domain extends into what would be a structured region in a tandem LIM construct. Indeed, in the LMO4_LIM1+2_•LDB1 structures [43], [44], LMO4_I94_ (which forms a backbone-backbone hydrogen bond with DEAF1_T414_ in the structure) forms an *intra*molecular backbone-backbone hydrogen bond that would preclude it from making an *inter*molecular hydrogen bond with a peptide binding-partner (**Fig. 6b**). Apart from that hydrogen bond, there are relatively few LMO4-DEAF1 contacts in this region of the complex (**Fig. 4c and e**). Although it is possible that the interaction between LMO4 and DEAF1 forms an atypical LIM-peptide interface, we think it is more likely that this region of the interface is an artefact resulting from construct design. Indeed, ^15^N-HSQC spectra show conservation of peaks from S208 and DEAF1_404–411_ for LMO4_LIM1+2_ and LMO4_LIM2_ complexes, but no conservation of peaks for DEAF1 residues that lie C-terminal to DEAF1_411_ (**Fig. S2** in **File S2**). Assuming that S208 is a good mimic of DEAF1_Q403_, there is high structural homology between DEAF1_403–410_ and LDB1_302–309_, despite poor sequence identity – only DEAF1_P408_ and LDB1_P307_ are identical in the two LMO4 partners (**Fig. 6**).

### The tandem LIM LMO4-DEAF1 interface

All characterised peptide-like LIM-interaction domains bind the equivalent faces of their target tandem LIM domains using two linear binding motifs roughly 8–10 residues long. The binding motifs are separated by a spacer of 1–9 residues [41]. We predict that the C-terminal portion of DEAF1_404–438_ will bind LMO4_LIM1_ in the same fashion as LDB1 and CtIP because our data shows the same key residues of LMO4 are implicated [19], [43], [44]. The mutagenic data for DEAF1 (**Fig. 1c**) indicate that two other portions of DEAF1_404–438_ may contribute to binding: DEAF1_419–421_ and DEAF1_434–438_. The former region forms a small hydrophobic cluster that is typical of peptide LIM-binding motifs [41] and, assuming a spacer of ∼5–6 residues, would be well placed to interact with the core binding site on LMO4_LIM1_. Spacers of this size are utilised by the Lhx3/4-binding domains of ISL1 and ISL2 [70], [71]. In contrast, the more distal residues (DEAF1_434–438_) are predicted to lie outside the LIM-binding motif. We have seen a similar effect upon mutation of some residues C-terminal of the LMO4-interaction domain in LDB1 [43] and CtIP [19]. These mutations might disrupt long-range interactions, but additional yeast-two hybrid data, in which we assessed the stability of one of these mutants through interaction with a C-terminal coiled-coil domain, indicates that these mutations destabilise the constructs in yeast cells (**Fig. S3** in **File S2)**.

Even with the identification of the short hydrophobic cluster DEAF1_I419/V420/L421_ as a likely binding motif, it is not possible to accurately predict the binding register of the LIM1-binding motif. For example, two possible registers based on those observed for LMO4-CtIP (where two residues are buried in the hydrophobic core of the LMO4_LIM1_ domain) and LMO4-LDB1 and related Lhx-ISL complexes (where a single residue is buried in the hydrophobic core of the LMO4_LIM1_ domain) are shown (**Fig. 6**). In the first model of binding (**Fig. 6d**), the side-chains of DEAF1_I419_ and DEAF1_L421_ are buried in the hydrophobic core of the protein, DEAF1_T422_ is correctly positioned to mimic LDB1_T323_ and CtIP_T671_, and DEAF1_K418_ mimics CtIP_K667_. In the second model of binding, which is based on the structure of Lhx3_LIM1+2_•ISL1_LBD_, the side-chain of V420 is buried (as is LDB1_I322_ or ISL1_V282_), and DEAF1_K418_ mimics LDB1_R320._ At this stage the mutational data does not allow us to distinguish the two models. Note that other binding modes are possible, and the angle between the LIM domains is hard to predict. These hinge/spacer regions vary considerably between LIM-peptide structures and in many cases show evidence of flexibility [70], [71], [73], [74], [75].

### Biological Implications

**Figure 7:**
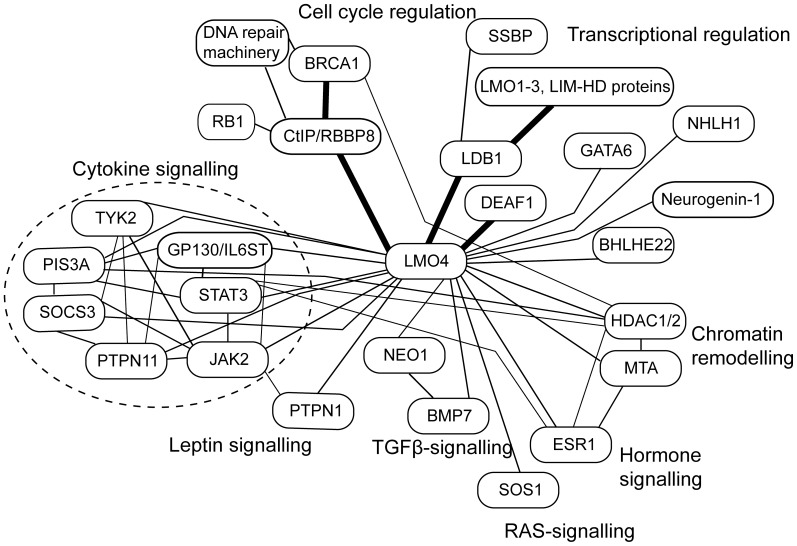
LMO4 is a protein-protein interaction network hub linking multiple cellular processes. Protein-protein interaction network assembled from data reported for mouse and human LMO4 proteins from the STRING protein-protein interaction database, plus additional papers cited in the introduction. Bold lines indicate protein-protein interactions that have been characterised structurally. Other lines indicate reported interactions that have different levels of evidence and some of these lines may represent indirect interactions. Proteins are loosely grouped into cellular processes.

Our data show that DEAF1, LDB1 and CtIP all bind to the same face on LMO4. If co-expressed and co-localised in a cell, they would therefore compete for binding to LMO4. LMO4 does not contain a nuclear localisation sequence (NLS), and is small enough (∼20 kDa) to passively diffuse into and out of the nucleus [27]. Nuclear localisation of LMO4 and other family members is likely to be facilitated by binding to partner proteins that contain NLSs such as the widely expressed LDB1 [17], whose competitive binding is important for cell specification in a range of tissue types [76], [77], [78]. Binding of LMO4 to DEAF1 facilitates nuclear localisation of DEAF1, apparently through modulation of the DEAF1 NES [52]. Simultaneously, DEAF1, which contains a nuclear localisation signal [53], likely facilitates nuclear localisation of LMO4. DEAF1 probably also sequesters LMO4 at gene regulatory elements via the DNA-binding SAND domain in DEAF1. However, whereas other LMO proteins appear to be predominantly nuclear, LMO4 can be found in either in the nucleus or distributed between the nucleus and cytoplasm [4], [26]. LMO4 can be palmitoylated at its C-terminal cysteine residue (C165) which facilitates retention of LMO4 in the cytoplasm and the endoplasmic reticulum [26]. This apparent ability of LMO4 to associate with membranes and to have a wider subcellular distribution provides a partial explanation for why LMO4 has a broader range of reported interaction partners than other LMO proteins. In terms of protein-protein interaction networks, LMO4 appears be a hub protein, connecting multiple signalling pathways including cytokine-, TGFβ-, leptin-, Ras- and hormone signalling (**Fig. 7**). LMO4 has strong links to transcriptional regulation, either through components of these pathways (e.g., via STAT3 and ESR1) or by mediating contacts with transcription factors (such as DEAF1, GATA6 and bHLH proteins), co-factor proteins (such as LDB1) and chromatin remodelling machinery. By regulating the expression of cyclin proteins [79], and through interaction with CtIP, LMO4 is likely to contribute to cell cycle regulation.

## Conclusions

DEAF1, Ldb1 and CtIP all appear to bind the same peptide-binding face on LMO4, suggesting that competitive binding for LMO4 and modulation of its subcellular localisation, likely plays a part in linking diverse cellular pathways. Subsequent disruptions to the normal expression patterns and subcellular localisation of LMO4 could therefore contribute to severe developmental abnormalities and breast cancer. Many partners of LMO4 contain putative intrinsically disordered regions that could contain LMO4-binding peptides. It will be interesting to determine if many of the other interaction partners of LMO4 also bind to the same surface.

## Supporting Information

File S1 **Zinc patch files for NMR structure determination in ARIA of the LIM2 domain from LMO4.** This zip file includes 8 compressed files: Zinc.param, wellordered.imp, topzinc.pro, topallhdg5.3_thz.pro, run.cns, generate_template.ino, generate_ion1.2.inp, and zinc.pdb.
(ZIP)

File S2 **Contains Supplemental data: Figure S1.** ^15^N-HSQC spectrum of LMO4_16–148_•L4-DEAF1; **Figure S2.** Overlay of ^15^N-HSQC spectra for LMO4_LIM1+2_•L4-DEAF1(black) and LMO4_LIM2_•DEAF1_404–418_(blue); and, **Figure S3.** The 434–36 triple alanine mutant of DEAF1 is destabilised in yeast compared to wild-type.
(PDF)
