# Supplementary material for: The Structure of an LIM-Only Protein 4 (LMO4) and Deformed Epidermal Autoregulatory Factor-1 (DEAF1) Complex Reveals a Common Mode of Binding to LMO4
Source: PLoS One. 2014 Oct 13;9(10):e109108. doi: 10.1371/journal.pone.0109108 (PMC4195752; doi:10.1371/journal.pone.0109108)
Supplement: File S2 — Contains Supplemental data: Figure S1. 15N-HSQC spectrum of LMO416–148•L4-DEAF1; Figure S2. Overlay of 15N-HSQC spectra for LMO4LIM1+2•L4-DEAF1(black) and LMO4LIM2•DEAF1404–418(blue); and, Figure S3. The 434–36 triple alanine mutant of DEAF1 is destabilised in yeast compared to wild-type. (PDF) [file pone.0109108.s002.pdf]

Supplementary Information S2

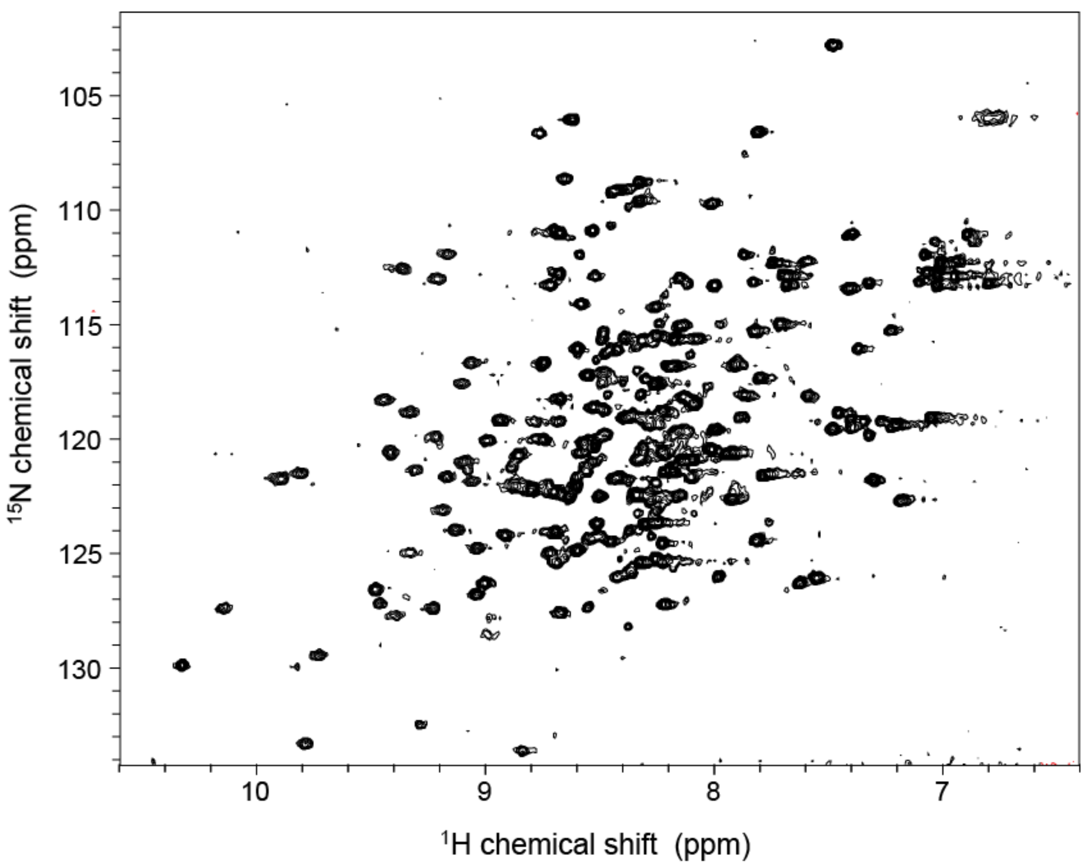

Fig S1. ^{15}N-HSQC spectrum of LMO4$_{16–148}$•L4-DEAF1. The protein (115 µM) was in 20 mM sodium acetate, 35 mM NaCl, 1 mM DTT (dithiothreitol) at pH 5.0. Spectra were recorded on a 600 MHz spectrometer at 310 K.

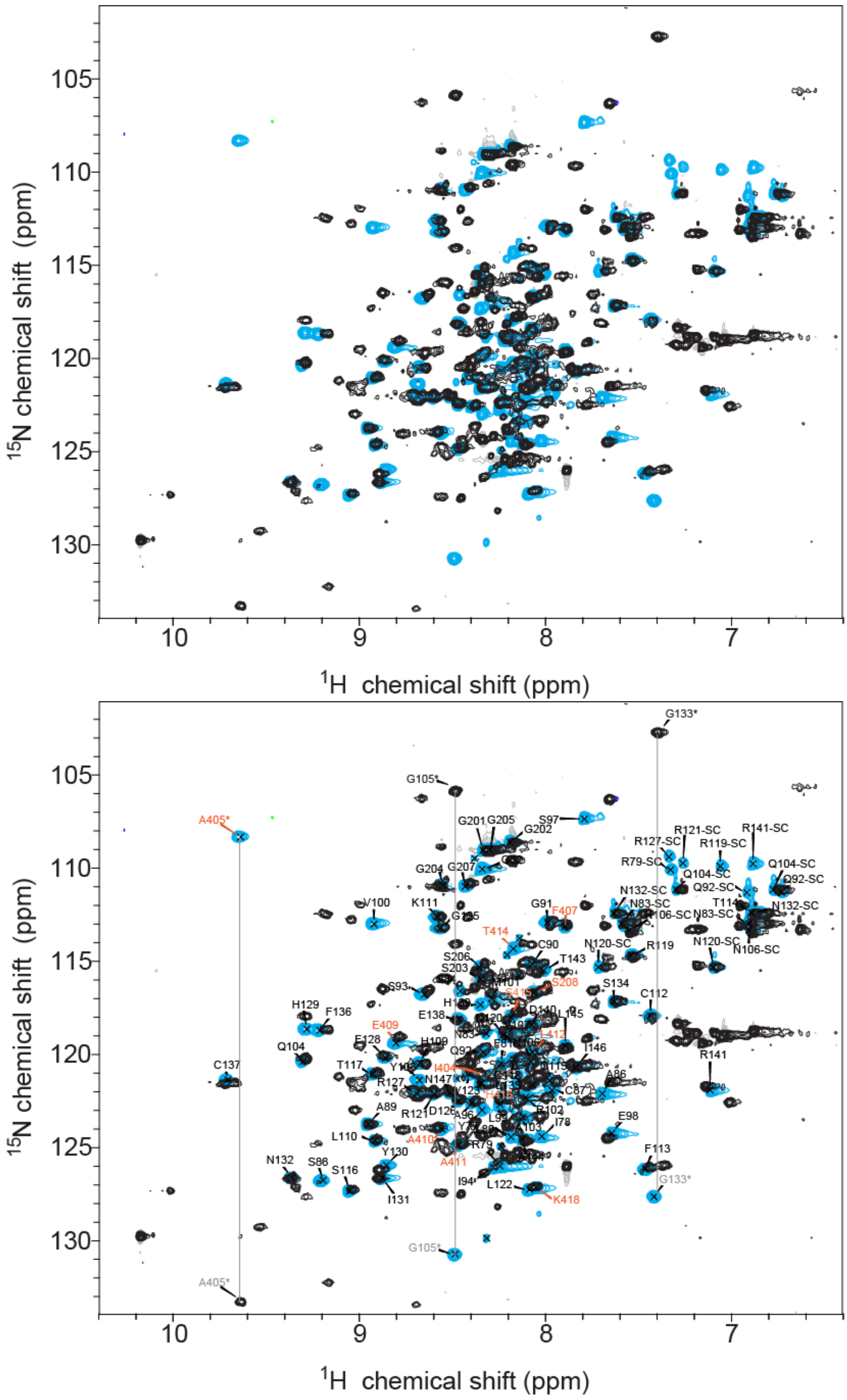

Fig S2. Overlay of ^{15}N-HSQC spectra for LMO4$_{LIM1+2}$•L4-DEAF1(black) and LMO4$_{LIM2}$•DEAF1$_{404–418}$(blue). The proteins were in 20 mM sodium acetate, 35 mM NaCl at pH 5.0.

The $LMO4_{LIM1+2}$•L4-DEAF1 samples contained 1 mM DTT. The $LMO4_{LIM2}$•$DEAF1_{404–418}$ sample also contained TCEP (tris(2-carboxyethyl)phosphine-HCl; 0.5 mM), chloramphenicol 34 µg/mL^{-1} and Complete EDTA-free protease (Roche; ~0.5 mM). Spectra were recorded on a 600 MHz spectrometer at 298 K. The top and bottom panels are identical except that the bottom panel includes labels showing peak assignments for $LMO4_{LIM2}$•$DEAF1_{404–418}$ [1] (BMRB: **18898**). Labels for S208 from the linker and DEAF1 are in orange. The peaks marked with an asterisks (*) were aliased for $LMO4_{LIM2}$•$DEAF1_{404–418}$ and the likely corresponding peaks in $LMO4_{LIM1+2}$•L4-DEAF1 are indicted (grey lines and labels).

There is substantial overlap of the spectrum of the smaller complex with that of the larger complex. Although the peaks for $LMO4_{LIM1+2}$•L4-DEAF1 have not been assigned, by inference peaks corresponding to residues from S208 and $DEAF1_{404–411,}$ and most residues in the $LMO4_{LIM2}$ domain from $LMO4_{LIM2}$•$DEAF1_{404–418}$ are recapitulated in the spectrum of the larger construct. Peaks corresponding to $DEAF1_{412–418}$ are either in crowded regions (making them impossible to distinguish) or appear to have shifted.

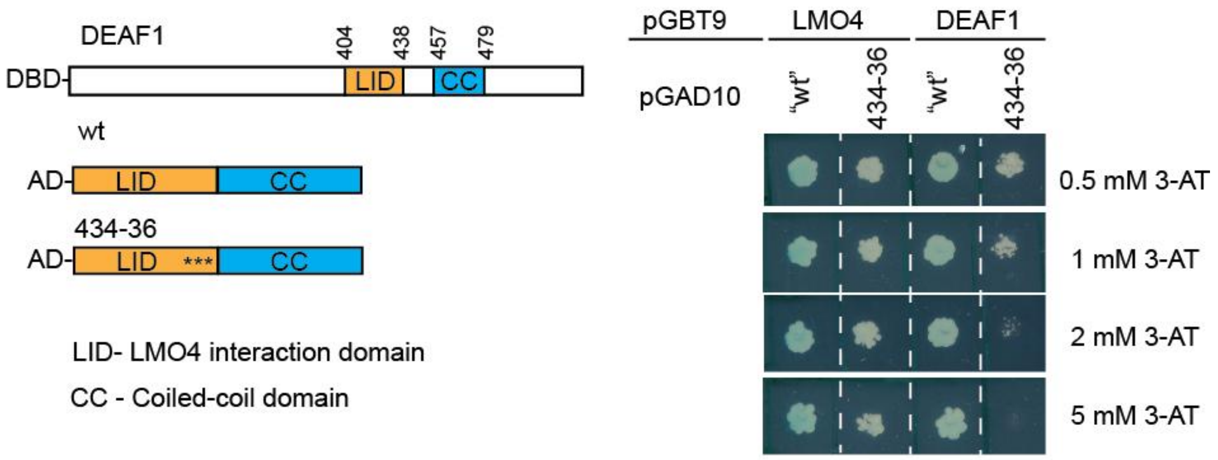

Fig S3. The 434–36 triple alanine mutant of DEAF1 is destabilised in yeast compared to wild-type. Yeast-two hybrid assays were used to compare the interaction of wt and mutant pGAD10-DEAF1$_{404–438_457–479}$ with pGBT9-LMO4 and pGBT9-DEAF1$_{45–566,}$ via the LMO4 interaction domain and coiled-coil domain of DEAF1, respectively. The 434–36 triple alanine mutant construct shows significant loss of self interaction through the coiled-coil domain under selection conditions that allow interaction with LMO4 (-L-W-H plus 0.5–5 mM 3-AT), suggesting that the mutant construct is destabilised and/or degraded compared to the wild-type ("wt"). Note that self interaction of DEAF1 via the coiled-coil domain was reported previously [2], and no growth of the mutant with LMO4 is seen under high stringency selection conditions (-L-W-A; Fig 1c).

References

[1] Joseph, S., Kwan, A. Y., Mackay, J., Cubeddu, L. & Matthews, J. M. (2014). Backbone and side-chain assignments of a tethered complex between LMO4 and DEAF-1. Biomol NMR Assign **8**, 141-4.

[2] Cubeddu L, Joseph S, Richard DJ, Matthews JM (2012) Contribution of DEAF1 structural domains to the interaction with the breast cancer oncogene LMO4. PLoS ONE 7: e39218.
